# Extraordinary rare type of rotational abnormality of the gut revealed in a 6-month-old infant presented with intussusception: a case report

**DOI:** 10.1093/bjrcr/uaag006

**Published:** 2026-05-23

**Authors:** Reza Jalli, Maryam Owrangi, Seyed Mohsen Dehghani, Seyed Sina Dehghani

**Affiliations:** Department of Radiology, Medical Imaging Research Center, Shiraz University of Medicine, Shiraz, Iran; Department of Radiology, Shiraz University of Medicine, Shiraz, Iran; Gastroenterohepatology Research Center, Shiraz Transplant Research Center, Nemazee Teaching Hospital, School of Medicine, Shiraz University of Medical Sciences, Shiraz, Iran; Department of Radiology, Shiraz University of Medicine, Shiraz, Iran

**Keywords:** malrotaion, radiology, pediatrics, rotational abnormality of the gut

## Abstract

Intussusception is a common cause of acute abdominal emergency in infants and is usually managed successfully with image-guided enema reduction. Failure of nonoperative reduction should prompt consideration of underlying anatomic abnormalities, including congenital intestinal rotational anomalies. We report a 6-month-old infant who presented with symptoms of intussusception. Abdominal ultrasonography demonstrated a classic target sign consistent with intussusception, without additional specific findings. Contrast enema reduction was unsuccessful and revealed an unexpected bowel configuration. Surgical intervention was subsequently performed. Postoperative imaging demonstrated abnormal bowel positioning consistent with a congenital rotational abnormality, characterized by an atypical, mirror-image distribution of the small bowel and colon that did not conform to classical definitions of malrotation or nonrotation. Recognition of atypical intestinal rotational patterns is clinically relevant in the evaluation of intussusception, particularly when nonoperative management fails. Awareness of these uncommon configurations may aid in accurate interpretation of imaging findings, timely surgical management, and improved characterization of congenital bowel anomalies.

## Introduction

Intussusception is the most common abdominal emergency in children under two years of age and is a leading cause of intestinal obstruction in this population.^1^ The condition involves telescoping of a segment of the bowel into an adjacent segment, typically the ileum into the colon.^2^ Hydrostatic or pneumatic enema reduction under imaging guidance is both diagnostic and therapeutic, with success rates exceeding 80% in uncomplicated cases.^3^^,^^4^ However, the presence of anatomical abnormalities such as intestinal malrotation or nonrotation can lead to atypical clinical and radiological presentations and contribute to reduction failure.^5^^,^^6^ Intestinal malrotation is a congenital anomaly caused by incomplete rotation and fixation of the midgut during embryogenesis, often diagnosed in neonates but occasionally detected incidentally in older infants.^7^ Intestinal malrotation encompasses a spectrum of congenital anomalies resulting from an incomplete or abnormal rotation of the primitive gut during embryonic development, leading to an atypical positioning of the small and large intestines within the abdominal cavity. Common forms include non-rotation and incomplete rotation, which can range from asymptomatic to life-threatening, particularly due to the risk of midgut volvulus. We present the case of a 6-month-old boy with intussusception and failed contrast enema reduction, where abnormal bowel positioning observed during fluoroscopy raised suspicion for an underlying rotational abnormality of the gut, subsequently confirmed by small bowel series.

## Case presentation

A 6-month-old boy with no significant medical history presented to the emergency department with a 6-hour history of intermittent crying, non-bilious vomiting, and two episodes of passage of bloody, mucoid stool (currant jelly stool). Physical examination revealed a mildly distended but soft abdomen. Vital signs were stable, and there were no signs of peritonitis.

Point-of-care abdominal ultrasound revealed a classic “target sign” consistent with intussusception. Apart from this finding, no other specific abnormalities were identified, and the liver, spleen, and superior mesenteric artery were in their normal anatomic position. The patient underwent water–soluble contrast enema under fluoroscopic guidance in order to confirm the diagnosis as well as reduction of intussusception.

**Figure 1.**
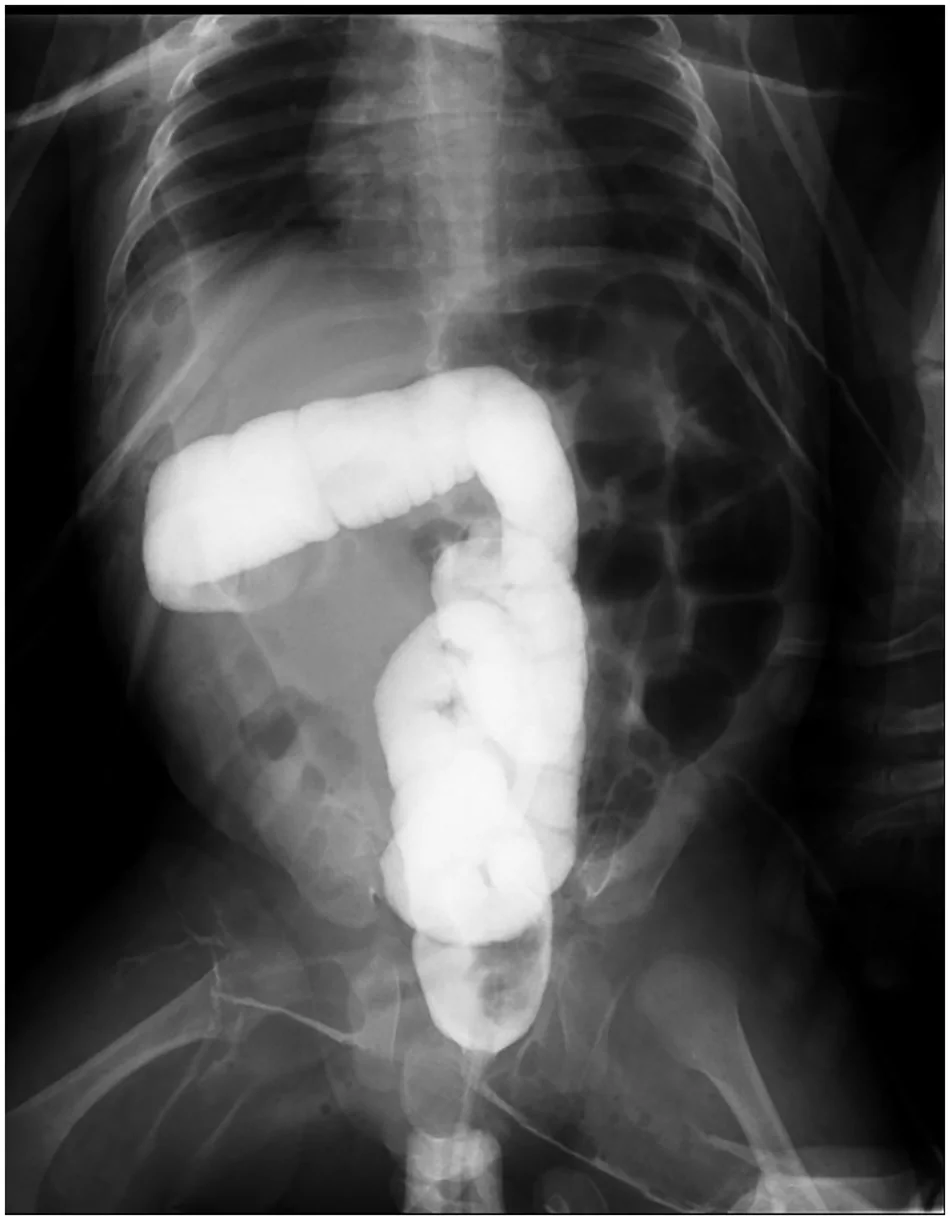
Failure of contrast to pass beyond the hepatic flexure indicates a complete obstruction, likely due to intussusception.

**Figure 2.**
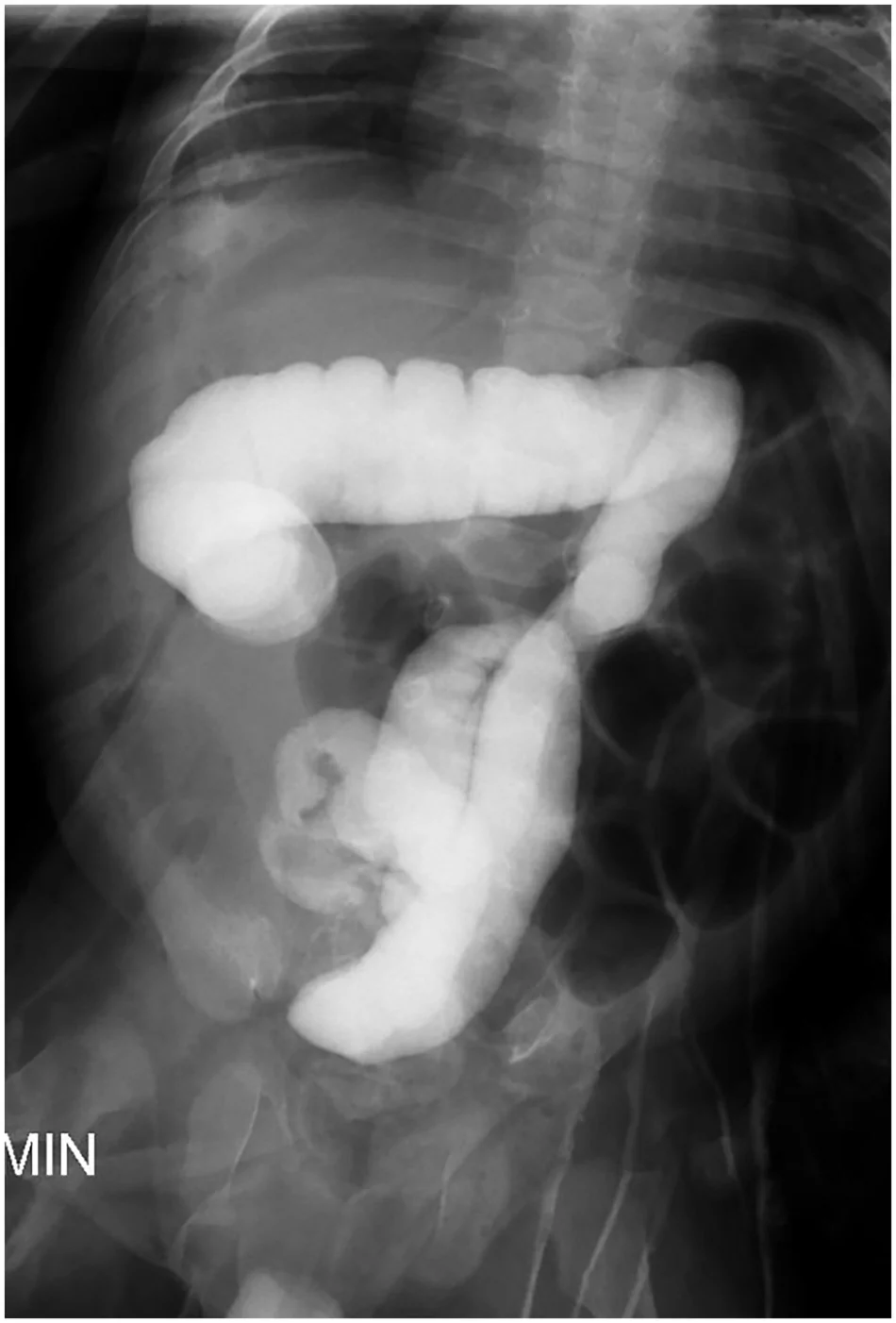
The small bowl loops show central and right-side location of colon associated with displacement of small bowel loops at left-side; these findings are suggestive of rotational abnormalities.

Contrast study disclosed redundant shape of sigmoid colon, central position of descending colon and complete blockage of right side of the large bowel below the level of hepatic flexure. Gas shadow of small bowel loops was predominantly visualized on the left side of abdomen (Figures 1 and 2).

Due to the failure of radiology measures to reduce intussusception (creating standard hydraulic pressure), the baby underwent laparotomy and surgical treatment. Findings during the surgery included a short ascending colon and ileocolic intussusception as well as central location of sigmoid and descending colon.

**Figure 3.**
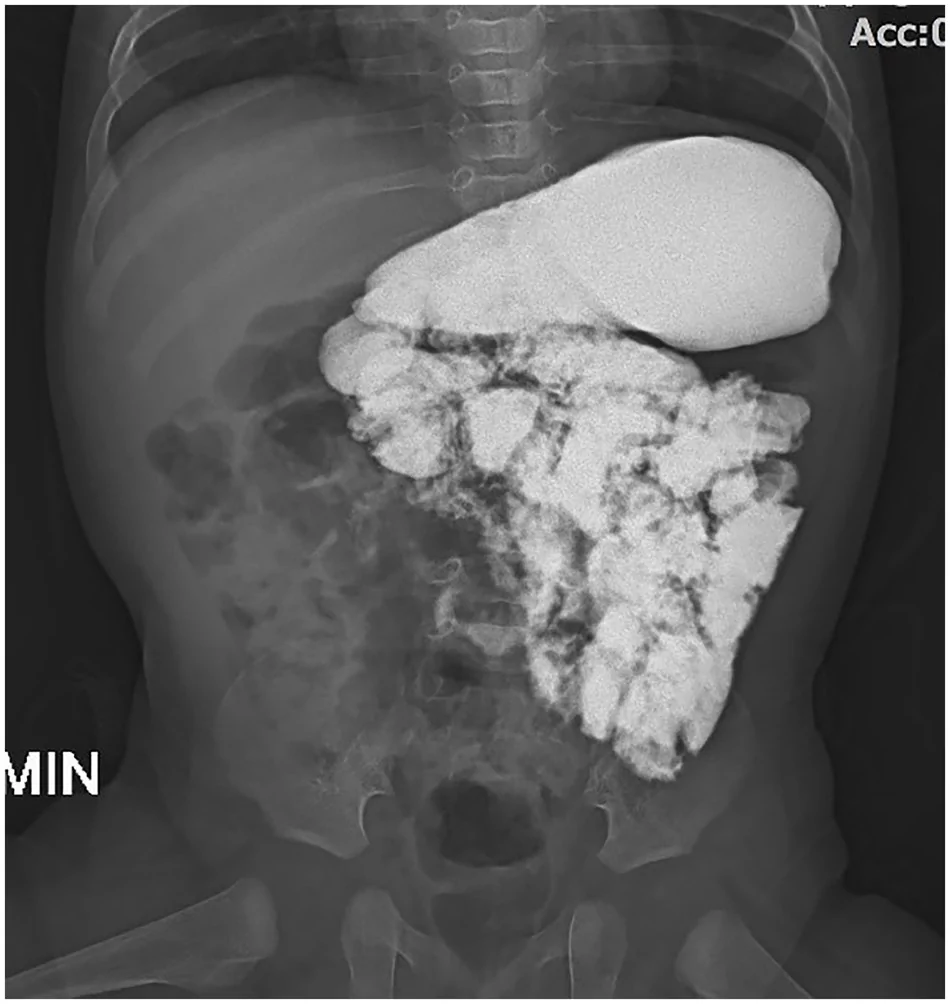
Early-phase small bowel follow-through shows clustering of contrast-filled small bowel loops in the left abdomen, suggestive of abnormal bowel positioning.

One week after surgery, a small bowel follow-through examination was performed for exclusion of midgut rotational abnormalities. In this study, duodenojejunal junction lying to the left of the midline, with normal configuration of small bowel loops and stomach (Figure 3). Chest X-ray and complementary abdominal ultrasound revealed no situs abnormality, organomegaly, or mass lesion.

## Discussion

This case highlights the diagnostic and procedural challenges posed by congenital anomalies in the context of intussusception. Intestinal malrotation refers to a spectrum of congenital anomalies resulting from abnormal rotation and fixation of the midgut during embryologic development. Normally, the midgut undergoes an approximately 270-degree counterclockwise rotation around the axis of the superior mesenteric artery, followed by normal fixation, resulting in the typical anatomic relationship of the duodenojejunal junction, small bowel, and colon.^7^ Disruption of this process produces a range of anatomic configurations rather than a single entity. Within this spectrum, nonrotation represents a specific variant in which normal midgut rotation fails to complete, commonly described as arrest after the initial phase of rotation, leading to abnormal separation of the small intestine and colon on opposite sides of the abdomen.^8^^,^^9^ Incomplete rotation, often referred to as classic malrotation, reflects partial progression of midgut rotation with abnormal fixation, which may result in a narrowed mesenteric base and increased susceptibility to midgut volvulus.^10^ Less frequent patterns, including reversed rotation and other atypical variants, have also been described and are best regarded as part of a broad continuum of congenital rotational abnormalities rather than discrete, rigid categories.^11^ Congenital rotational abnormalities of the gut may remain clinically silent or manifest with a wide spectrum of complications, largely determined by the degree of abnormal rotation and fixation of the midgut. The most severe and well-recognized complication is midgut volvulus, which results from a narrowed mesenteric base and can rapidly progress to bowel ischemia and necrosis, constituting a surgical emergency.^12^ Other potential complications include intermittent or chronic intestinal obstruction, internal herniation, abnormal peritoneal fixation bands, and nonspecific or recurrent gastrointestinal symptoms that may delay diagnosis.^13^ Beyond volvulus, abnormal bowel orientation and fixation have also been implicated in the development of intussusception, particularly in infants and young children. Altered anatomic relationships between bowel segments may disrupt normal peristaltic coordination, predisposing the intestine to telescoping and, in some cases, contributing to failure of nonoperative enema reduction.^14^^,^^15^

In the present case, the intestinal configuration differed from the classical anatomic descriptions of well-recognized rotational patterns. Although abnormal positioning of the bowel was evident, the relationship between the small bowel and colon did not conform to the typical arrangements described in standard nonrotation or classic malrotation. Instead, the bowel demonstrated an unusual mirror-image distribution, with the colon predominantly located on one side of the abdomen and the small bowel clustered on the opposite side, in a pattern that cannot be readily explained by arrest at a single, defined stage of midgut rotation. From an embryologic perspective, this finding suggests a variant pattern of rotational development, likely reflecting an atypical degree or direction of midgut rotation rather than a textbook form of rotational arrest.

Recognition of abnormal bowel positioning on imaging should prompt consideration of underlying congenital anomalies and early surgical referral. The use of fluoroscopy not only provides therapeutic opportunity but also serves as a valuable diagnostic tool in identifying anatomical variants during enema reduction attempts.

## Conclusion

Congenital intestinal rotational anomalies may present with non-classical and heterogeneous anatomic patterns that do not always conform to standard definitions. In the setting of intussusception, particularly when nonoperative reduction is unsuccessful, recognition of atypical bowel configurations is clinically relevant, as underlying rotational variants may influence both presentation and management. Awareness of these uncommon patterns can facilitate appropriate interpretation of imaging findings, timely surgical decision-making, and more accurate characterization of congenital bowel anomalies.

## Learning points

- Failed enema reduction in intussusception should raise suspicion for underlying anatomical anomalies such as intestinal malrotation or nonrotation.
- Recognition of abnormal bowel positioning during fluoroscopic procedures can provide vital diagnostic clues and guide early surgical intervention.
- Postoperative imaging, such as small bowel follow-through, is essential to confirm congenital rotational abnormalities and inform long-term clinical management.
- Awareness of these rare anomalies ensures appropriate diagnosis and treatment, especially in cases deviating from the typical presentation.
